# Uncertainty in the positioning of patients receiving treatment for brain metastases and wearing surgical mask underneath thermoplastic mask during COVID‐19 crisis

**DOI:** 10.1002/acm2.13279

**Published:** 2021-05-24

**Authors:** Hideharu Miura, kazunari Hioki, Shuichi Ozawa, Kenji kanemoto, Minoru Nakao, Yoshiko Doi, Masahiko Kenjo, Yasushi Nagata

**Affiliations:** ^1^ Hiroshima High‐Precision Radiotherapy Cancer Center Hiroshima 732‐0057 Japan; ^2^ Department of Radiation Oncology Institute of Biomedical & Health Sciences Hiroshima University Hiroshima 739‐8511 Japan

**Keywords:** COVID‐19, image guidance, immobilization, patient positioning error, surgical mask

## Abstract

Thermoplastic masks, used along with surgical masks, enable immobilization methods to reduce the risk of infection in patients undergoing intracranial stereotactic radiosurgery and stereotactic radiotherapy (SRS/SRT) during the COVID‐19 crisis. The purpose of this study was to investigate the feasibility of thermoplastic mask immobilization with a surgical mask using an ExacTrac system. Twelve patients each with brain metastases were immobilized using a thermoplastic mask and a surgical mask and only a thermoplastic mask. Two x‐ray images were acquired to correct (XC) and verify (XV) the patient’s position at a couch angle of 0°. Subsequently, the XC and XV images were acquired at each planned couch angle for non‐coplanar beams. When the position errors were detected after couch rotation for non‐coplanar beams, the errors were corrected at each planned couch angle until a clinically acceptable tolerance was attained. The position errors in the translational and rotational directions (vertical, lateral, longitudinal, pitch, roll, and yaw) were retrospectively investigated using data from the ExacTrac system database. A standard deviation of XC translational and rotational position errors with and without a surgical mask in the lateral (1.52 vs 2.07 mm), longitudinal (1.59 vs 1.87 mm), vertical (1.00 vs 1.73 mm), pitch (0.99 vs 0.79°), roll (1.24 vs 0.68°), and yaw (1.58 vs 0.90°) directions were observed at a couch angle of 0°. Most of patient positioning errors were less than 1.0 mm or 1.0° after the couch was rotated to the planned angle for non‐coplanar beams. The overall absolute values of the translational and rotational XV position errors with and without the surgical mask were less than 0.5 mm and 0.5°, respectively. This study showed that a thermoplastic mask with a surgical mask is a feasible immobilization technique for brain SRS/SRT patients using the ExacTrac system.

## 1 INTRODUCTION

Stereotactic radiosurgery and stereotactic radiotherapy (SRS/SRT) are well‐established techniques for high‐precision treatment of intracranial benign and malignant lesions in 1–5 fractions. The accuracy of treatment planning and delivery is highly dependent on accurate patient positioning and immobilization to achieve local tumor control and spare normal tissue. Several stereotactic systems have been used to immobilize these patients accurately during treatment.^1^, ^2^, ^3^, ^4^, ^5^, ^6^, ^7^, ^8^ Conventionally, invasive immobilization, such as metal frames or rings fixed to the patient’s skull, has been used for patient immobilization and target localization in the SRS/SRT treatment of intracranial lesions.^4^ Non‐invasive (frameless) SRS/SRT, such as thermoplastic mask systems, has become a standard immobilization method owing to the advent of image‐guided radiotherapy (IGRT) systems. One of the advanced IGRT systems, the ExacTrac system (BrainLAB A.G., Heimstetten, Germany), is an integration of an infrared (IR)‐based optical positioning system and a radiographic kilovolt (kV) x‐ray imaging system that accurately determines patient positions and makes online corrections.

Pneumonia resulting from an unknown cause was reported to the World Health Organization (WHO) country office in Wuhan on December 31, 2019. The WHO named this unknown disease “coronavirus disease 2019” (COVID‐19). COVID‐19 has rapidly spread worldwide and is a global health concern. Several publications have provided recommendations and measures for radiation oncology clinics to prevent infection during the COVID‐19 pandemic.^9^, ^10^, ^11^, ^12^, ^13^, ^14^ Effective protective methods are required to prevent the spread of COVID‐19 during radiation implementation. Immobilization devices for head and neck patients should be cleaned using a sanitizer, as they are in contact with the patient's mouth/nose. Wearing a surgical mask is a low‐cost and remarkably effective intervention to reduce the spread of the virus in people infected with COVID‐19. Patients should wear the mask during the treatment or an additional face shield must be placed properly in cases where the patient has to take off the mask during treatment, such as a personalized head and neck immobilization mask. In our institution, patients undergoing treatments involving the head and neck area wear the surgical mask underneath the thermoplastic mask during treatment. We inquired whether wearing the surgical mask underneath the thermoplastic mask by patients receiving SRS/SRT leads to an inaccurate patient position.

In this study, we investigated the patient position errors for brain SRS/SRT‐treated patients with and without a surgical mask underneath the thermoplastic mask using daily pretreatment imaging. This study analyzes patient positioning corrections that are performed during treatments using the ExacTrac system, which consists mainly of two procedures: kV x‐ray images for initial patient setup and position verification.

## 2 MATERIAL AND METHODS

### 2.A Patients and simulation section

Between February 2020 and November 2020, 24 patients with intracranial lesions treated with SRS/SRT at our institution were included in this study. Twelve selected patients were treated with the surgical mask and thermoplastic mask combinatory design from June 2020. To compare the immobilization accuracy, 12 selected patients wearing only thermoplastic masks were selected prior to these preventive measures.

### 2.B Computed tomography (CT) simulation and treatment planning

**Fig. 1:**
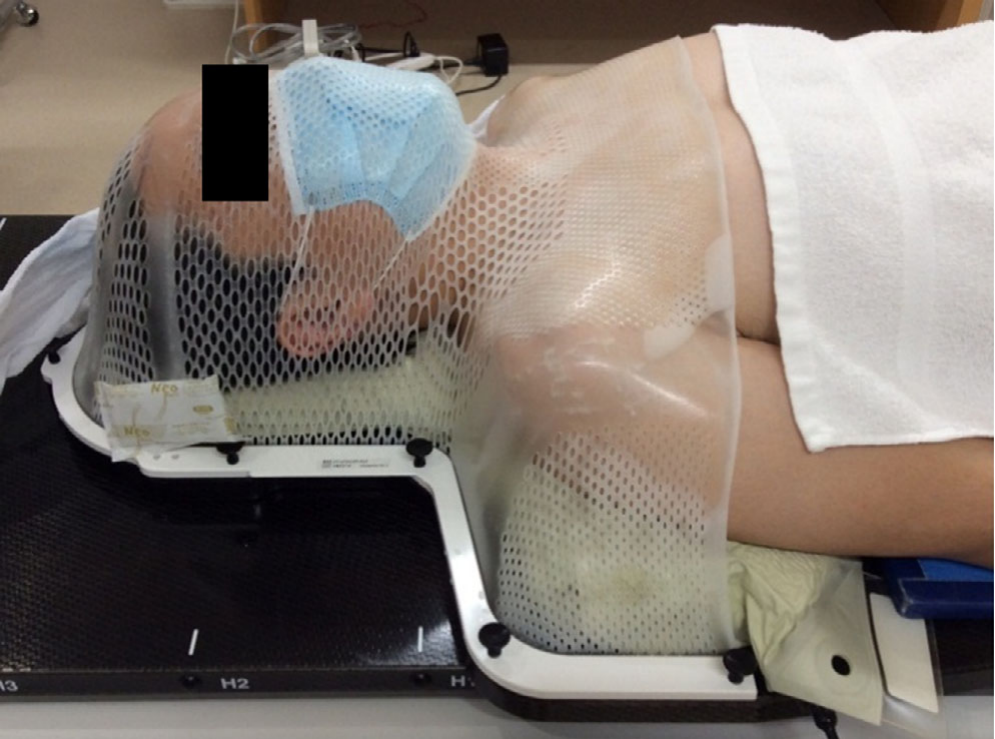
Patient wearing thermoplastic mask with a surgical mask.

A noninvasive thermoplastic mask (CIVCO Medical Solutions, Kalona, IA, USA) was used to immobilize all patients undergoing SRS/SRT for intracranial lesions. Patients wore surgical masks to avoid the risk of infection. A thermoplastic mask was formed on a patient who wore a surgical mask (Fig. 1). Patients wore the surgical mask upside down to prevent pain from the noise wire. The immobilization mask was carefully made to avoid space around the surgical mask. For patients, a simulation computed tomography (CT) scan (Optima CT 580 W; GE Healthcare, Milwaukee, WI, USA) was performed after molding. The CT scan parameters were set as follows: x‐ray tube voltage, slice thickness, and field of view values were 120 kV, 1.25 mm, and 500 mm, respectively, and the mAs value was determined by an auto‐exposure control function. All CT data were exported to the Eclipse (version 13.5, Varian Medical Systems, Palo Alto, CA, USA) treatment planning system (TPS), commissioned through a TrueBeam STx (Varian Medical Systems, Palo Alto, CA) linear accelerator. Gross tumor volume (GTV) was defined as an abnormality on T1‐weighted MRI with Gd. The clinical target volume was equal to that of the GTV. The planning target volume (PTV) was generated by adding a 1‐mm margin from the GTV. The dose was prescribed with an 80% isodose line covering the PTV. Volumetric modulated arc therapy (VMAT) with beam energies of 6 or 10 Megavoltage (MV) (flattening filter‐free (FFF) mode) was used for all patients. An axial coplanar arc of 360 ° and two to three non‐coplanar arcs of 180° were used for VMAT.

### 2.C Patient setup procedure

First, the patient was manually positioned at the isocenter using a room laser attached to the wall at a couch angle of 0°. This step only used the three translational axes of the 6 degree‐of‐freedom (6D) couch. Second, two radiographic kV x‐ray images were acquired and matched with the reference digitally reconstructed radiographs (DRRs) from the simulation CT images generated by the ExacTrac software at the cough gantry at 0°. The ExacTrac system was used for rigid image fusion with the bone anatomy, and it calculated the necessary translational and rotational 6D couch shift values for moving the patient to the isocenter position. The couch position was corrected using the IR guidance system by monitoring the IR reflective markers attached to the cranial positioning array (BrainLAB A.G., Heimstetten, Germany). Otherwise, shift values were applied by moving a robotic couch capable of 6D translational and rotational shift correction. After moving the couch shifts, the second set of radiographic kV x‐ray images was acquired to verify the patient’s position. The final patient translational and rotational positions were within a tolerance of 0.5 mm and 0.5°, respectively, and then the treatment delivery was started. X‐ray images at each planned couch angle for non‐coplanar beams were acquired to correct the patient’s position prior to beam delivery. This process was repeated until all shift values based on the patients’ anatomy matched within tolerance with x‐ray imaging.

### 2.D Data analysis

XC and XV position errors were defined as the calculated shift values based on the first and last two x‐ray images at each couch angle for treatment. XC and XV 6D translational and rotational (lateral, longitudinal vertical, pitch, roll, and yaw) position errors were extracted from the ExacTrac system database for each patient with all treatment beams. We calculated the mean and standard deviation of the translational and rotational XC and XV position values to evaluate the patient positioning error. The Wilcoxon signed‐rank test with was performed with *P* < 0.05 taken as the criterion for statistical significance to compare the translational and rotational position error with and without a surgical mask using the software R (version 3.5.2; www. r‐project.org).

## 3 RESULTS

**Fig. 2:**
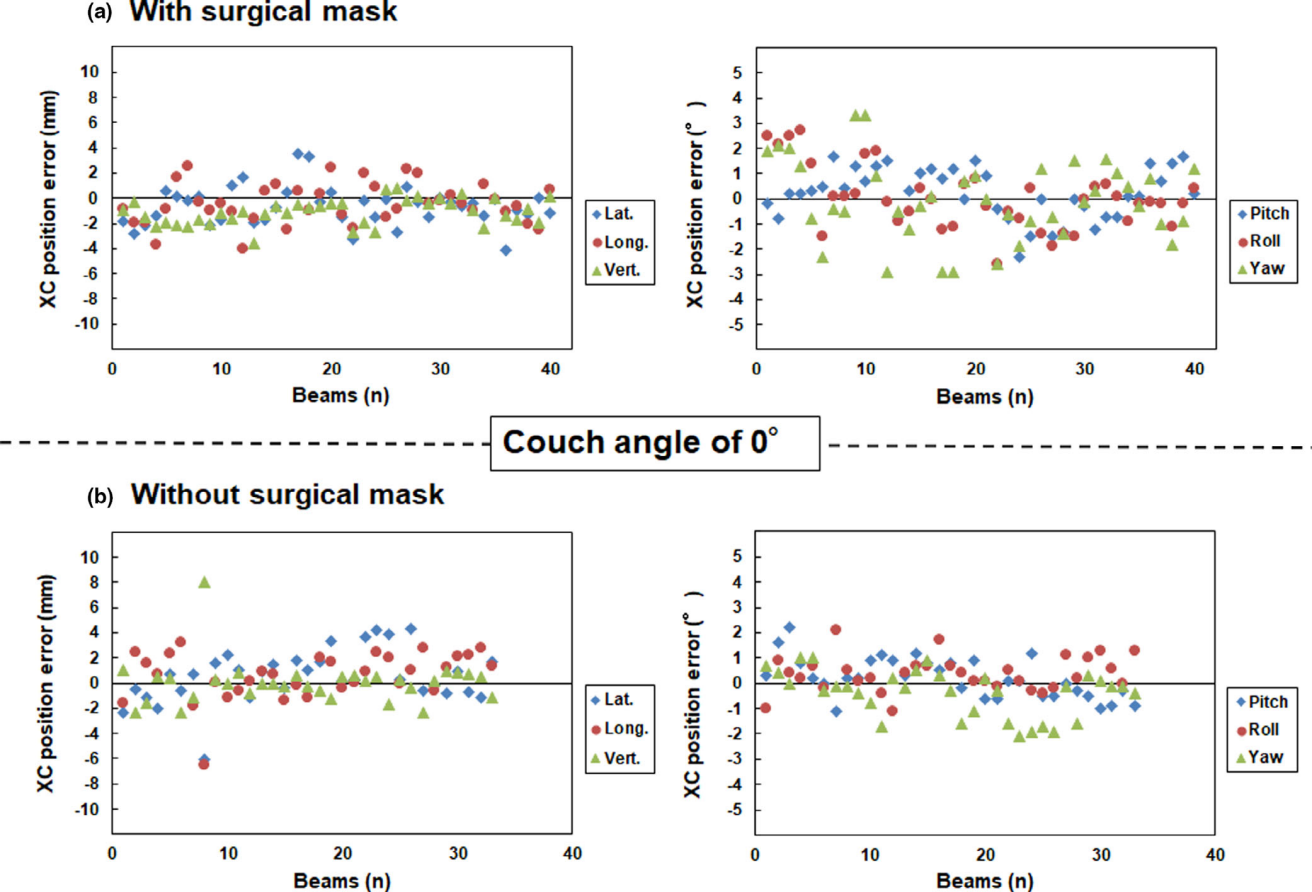
Translational (left panel) and rotational position (right panel) errors (a) with and (b) without surgical masks at the couch angle of 0° on the XC verification, respectively. Note: Y axis range is from −10.0 to 10.0 mm.

**Fig. 3:**
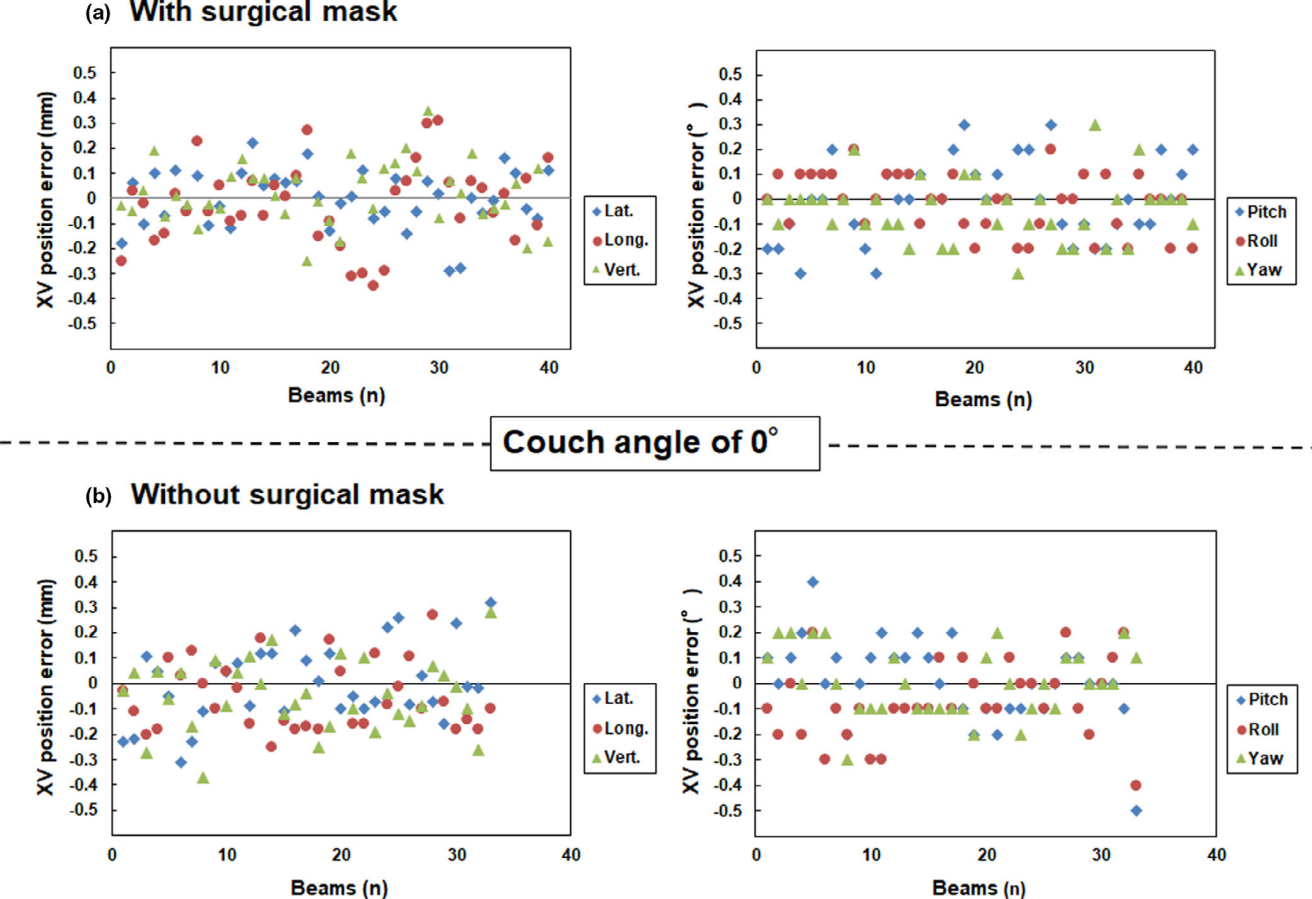
Translational (left panel) and rotational position (right panel) errors (a) with and (b) without surgical masks at the couch angle of 0° on the XV verification, respectively. Note: Y axis range is from −0.5 to 0.5 mm.

Fig. 2 and 3 show the translational and rotational XC and XV position errors with and without surgical masks at a couch angle of 0°, respectively. Table 1 shows the mean and standard deviation values calculated from the translational and rotational XC and XV position errors at a couch angle of 0°. A standard deviation of translational and rotational XC position error with and without a surgical mask in the lateral (1.52 vs 2.07 mm), longitudinal (1.59 vs 1.87 mm), vertical (1.00 vs 1.73 mm), pitch (0.99 vs 0.79°), roll (1.24 vs 0.68°), and yaw (1.58 vs 0.90°) directions were observed at a couch angle of 0°, respectively.

**Table 1:** Position errors with and without a surgical mask at a couch angle of 0°.

Index	XC position error		*P‐value*	XV position error		*P‐value*
	With mask	Without mask		With mask	Without mask	
Lat (mm)	−0.65 ± 1.52 (−4.12–3.48)	0.57 ± 2.07 (−6.05–4.36)	0.020	0.00 ± 0.11(−0.29–0.22)	0.0 ± 0.15 (−0.31–0.32)	0.682
Lng (mm)	−0.42 ± 1.59 (−3.98–2.51)	0.60 ± 1.87 (−6.54–3.19)	0.021	−0.02 ± 0.16 (−0.35–0.31)	−0.05 ± 0.13 (−0.25–0.27)	0.570
Ver (mm)	−1.09 ± 1.00 (−3.59–0.77)	−0.08 ± 1.73 (−2.39–8.05)	0.001	0.02 ± 0.12 (−0.25–0.35)	−0.05 ± 0.14 (−0.37–0.28)	0.547
Pitch (°)	0.21 ± 0.99 (−2.30–1.70)	0.21 ± 0.79 (−1.10–2.20)	0.545	−0.01 ± 0.15 (−0.30–0.30)	0.01 ± 0.16 (−0.50–0.40)	0.547
Roll (°)	0.02 ± 1.24 (−2.60–2.70)	0.37 ± 0.68 (−1.10–2.10)	0.315	−0.01 ± 0.12 (−0.20–0.20)	−0.07 ± 0.15 (−0.40–0.20)	0.124
Yaw (°)	−0.06 ± 1.58 (−2.90–3.30)	−0.39 ± 0.90 (−2.10–1.00)	0.264	0.04 ± 0.12 (−0.30–0.30)	0.00 ± 0.13 (−0.30–0.20)	0.151

Values are shown as mean ± 1 standard deviation (range).

Abbreviations: Lat, lateral; Lng, longitudinal; Ver, vertical; XC, x‐ray correction; XV, x‐ray verification.

**Fig. 4:**
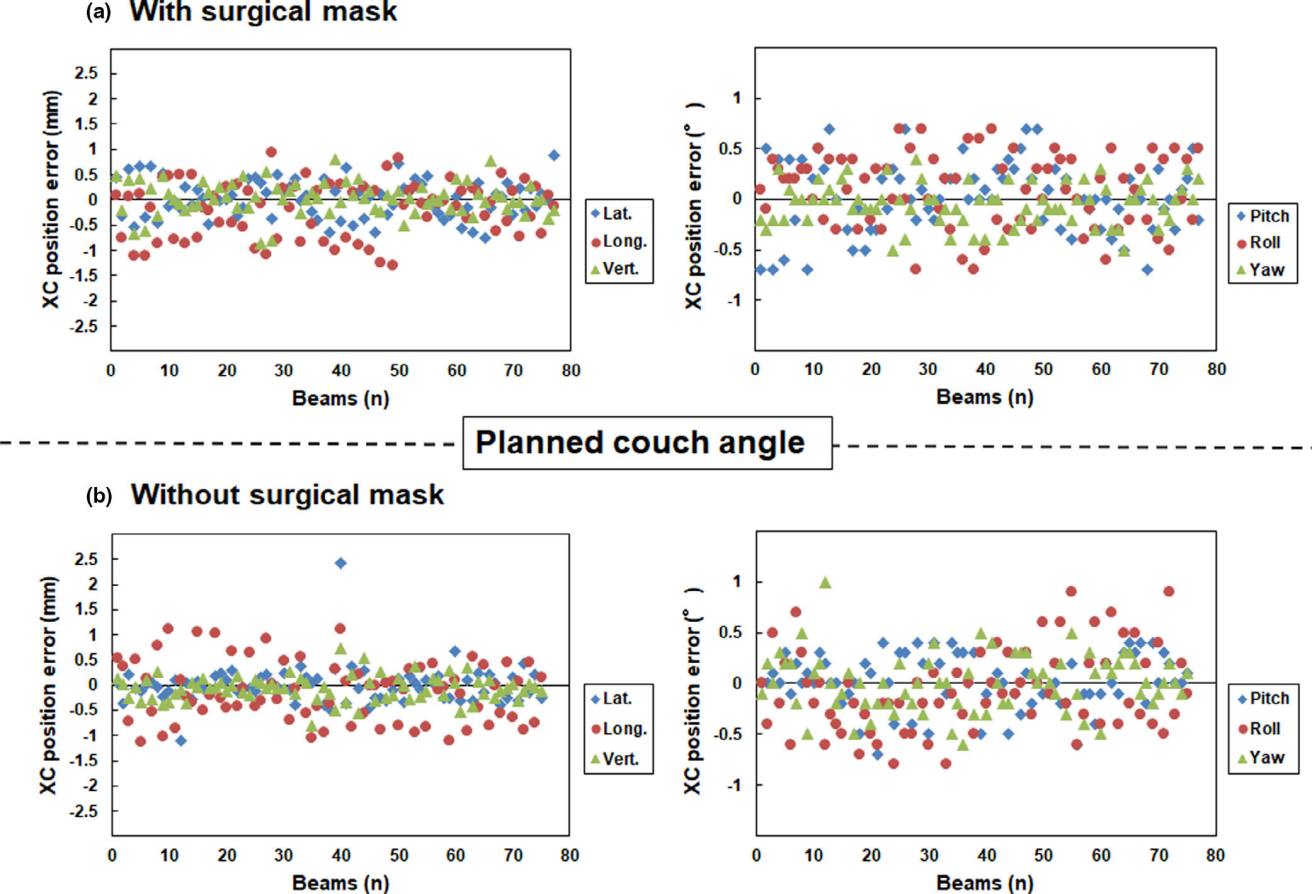
Translational (left panel) and rotational position (right panel) errors (a) with and (b) without surgical masks at the planned couch angle for non‐coplanar beams on the XC verification, respectively. Note: Y axis range is from −2.5 to 2.5 mm.

**Fig. 5:**
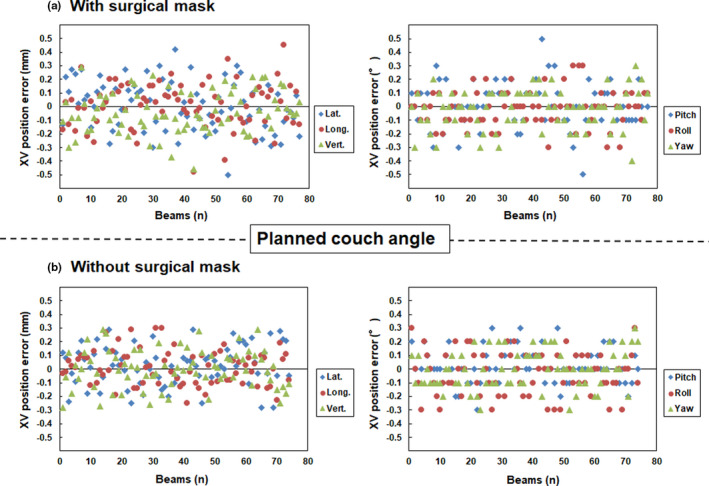
Translational (left panel) and rotational position (right panel) errors (a) with and (b) without surgical masks at the planned couch angle for non‐coplanar beams on the XV verification, respectively. Note: Y axis range is from −0.5 to 0.5 mm.

Fig. 4 and 5 show the translational and rotational XC and XV position errors, respectively, with and without surgical masks at each planned couch angle for non‐coplanar beams. Table 2 shows the mean and standard deviation values calculated from the translational and rotational XC and XV position errors at each planned couch angle for non‐coplanar beams. A standard deviation of translational and rotational XC position error with and without a surgical mask in the lateral (0.37 vs 0.39 mm), longitudinal (0.54 vs 0.59 mm), vertical (0.31 vs 0.25 mm), pitch (0.35 vs 0.26°), roll (0.35 vs 0.40°), and yaw (0.21 vs 0.28°) directions were observed at each planned couch angle for non‐coplanar beams, respectively. The percentages of translational and rotational XC position error with and without surgical mask within 1.0 mm or 1° at each planned couch for non‐coplanar beams were lateral (100.0 vs 97.3%), longitudinal (93.5 vs 89.3%), vertical (100.0% vs 100.0%), pitch (100.0% vs 100.0%), roll (100.0 vs 100.0%), and yaw (100.0 vs 98.7%) directions, respectively.

**Table 2:** Position errors with and without a surgical mask at each planned couch angle for non‐coplanar beams.

Index	XC position error		*P‐value*	XV position error		*P‐value*
	With mask	Without mask		With mask	Without mask	
Lat (mm)	0.02 ± 0.37 (−0.75–0.88)	0.00 ± 0.39 (−1.09 ‐ 2.42)	0.562	0.01 ± 0.18 (−0.50–0.42)	0.03 ± 0.15 (−0.28–0.29)	0.802
Lng (mm)	−0.17 ± 0.54 (−1.30–0.94)	−0.12 ± 0.59 (−1.13–1.12)	0.662	0.01 ± 0.16 (−0.48–0.45)	0.01 ± 0.12 (−0.25–0.30)	0.258
Ver (mm)	0.03 ± 0.31 (−0.86–0.80)	−0.06 ± 0.25 (−0.81–0.73)	0.053	−0.04 ± 0.15 (−0.46–0.29)	0.0 ± 0.14 (−0.28–0.29)	0.147
Pitch (°)	0.03 ± 0.35 (−0.70–0.70)	0.01 ± 0.26 (−0.70–0.40)	0.797	0.01 ± 0.16 (−0.50–0.50)	0.03 ± 0.13 (−0.30–0.30)	0.455
Roll (°)	0.09 ± 0.35 (−0.70–0.70)	−0.07 ± 0.40 (−0.80–0.90)	0.023	−0.01 ± 0.13 (−0.30–0.30)	−0.03 ± 0.15 (−0.30–0.30)	0.540
Yaw (°)	−0.05 ± 0.21 (−0.50–0.40)	0.01 ± 0.28 (−0.60–1.00)	0.167	−0.02 ± 0.14 (−0.40–0.30)	0.00 ± 0.14 (−0.30–0.30)	0.408

Values are shown as mean ± 1 standard deviation (range).

Abbreviations: Lat, lateral; Lng, longitudinal; Vrt, vertical; XC, x‐ray correction; XV, x‐ray verification.

Translational and rotational XV position errors with and without surgical masks were less than 0.5 mm and 0.5° in all cases, respectively. The Wilcoxon signed‐rank test showed no statistically significant differences in XV position errors with and without surgical masks. (*P* > 0.05).

## 4 DISCUSSION

In this study, we investigated patient position errors in SRS/SRT with and without a surgical mask underneath the thermoplastic mask. Our analysis results showed that XC position errors at the couch angle of 0° were <5.0 mm, except for one case. Keeling et al. reported that the mean translational XC shifts ranged from −2.5 to 2.5 mm with the Brainlab localizer, while rotational shifts were found to be usually less than 1°.^2^ They found uncertainties as large as 5.84 mm and 2.4° for translational and rotational shifts, respectively. They concluded that the translational couch position uncertainties were dependent on the couch angle. Tanaka et al. reported that the mean translational XC shifts for each of 70 treatment plans nearly ranged between −5.0 and 5.0 mm. These SD values are significantly larger than 1.0 mm in SRS/SRT with the frameless 6D ExacTrac system.^3^ They reported that the position error might be caused by both the patient’s intra‐fractional motion and the couch angle dependence of the couch position accuracy. The patient movement due to the couch rotation would most likely result in a random error. They concluded that either IGRT should be employed at each planned couch angle to correct patient position error, or a PTV margin should be enlarged by more than 2.0 mm in the clinically acceptable tolerance. Our analysis results showed that most of the patient position errors were <1.0 mm or 1.0° after rotating a couch to the planned angle for non‐coplanar beams because XC has been corrected at couch angle 0 before performing XC at other couch angles. This indicates that the intra‐factional‐patient motion was very small during each beam delivery. These values include the couch position accuracy. These position errors might not cause a major problem, as the patient setup could be quickly corrected at each planned couch angle for non‐coplanar beams using the ExacTrac system.

Clinical and medical physics tasks with no historical precedent have been introduced because of the COVID‐19 outbreak. Thus, it is useful to review some considerations and recommendations for the prevention of nosocomial transmission in oncology clinics.^9^, ^10^, ^11^, ^12^, ^13^, ^14^ Wei et al. studied the patient setup accuracy for cranial and head‐and‐neck patients immobilized between the control group with thermoplastic immobilization mask alone and the experimental group with a surgical mask underneath the thermoplastic immobilization mask, by comparing the cone‐beam computed tomography (CBCT) 6D shift/rotation results. The Student’s t‐test showed no statistical difference between the two groups on any three translational and three rotational motion shifts.^9^ Ohira et al. compared the intrafractional patient setup error with and without a mask, a bite block (BB), during fractionated intracranial stereotactic irradiation (STI) with thermoplastic masks and concluded that the required accuracy for STI can be maintained even without BB.^5^ Their strategy for image registration was to correct patient position using CBCT, and bony registration was performed using the MV images at each planned couch angle for non‐coplanar beams. The CBCT requires longer acquisition time with higher imaging dose, and MV imaging is associated with poor image contrast characteristics owing to the dominance of Compton interactions among photons in the MV spectrum. ExacTrac system is a tool for quickly positioning and acquiring images for IGRT of the patient and could slightly shorten the hospital stay time.^14^

Previous studies have reported that immobilization accuracy affects intra‐fractional patient motion during treatment, and a single image acquired prior to treatment is not sufficient to monitor patient motion.^6^ The duration of time spent by patients at the clinic or treatment room should be kept at a minimum to reduce the risk of COVID‐19 infection.^14^ After image guidance, the beam‐on‐time for each delivery beam is approximately 1–2 min. In addition, the ExacTrac system is a useful image‐guided option for patients with brain SRT, even at different couch rotations. Therefore, we believe that intra‐fractional patient motion using a thermoplastic mask system with a surgical mask is small. We are aware that a thermoplastic mask with a surgical mask forces patients to keep their mouth closed during treatment, which is uncomfortable. Full‐head masks can be cut open around the mouth to improve patient comfort. Previously published data showed that open‐face masks, in which the superior and inferior edges were pressed against the forehead and chin, can provide immobilization within 2.0 mm.^7^, ^8^ We believe that immobilizing these two points is stable and effective, even if there is a space between the thermoplastic mask and the mouth. Some patients may claim that a thermoplastic mask with a surgical mask is uncomfortable. We will cut the thermoplastic mask around the mouth as a solution.

Our study was limited to a small cohort of patients at the beginning of the COVID‐19 crisis. We will not further investigate the impact of a surgical mask on patient positioning error in a large population because we hope the infection will cease to spread further. We attempted to offer a solution to protect patients from COVID‐19 infection without compromising oncologic outcomes. In the future, we plan to report our study of clinical outcomes and toxicity in patients with brain metastases using a thermoplastic mask with a surgical mask. We hope this manuscript will be useful for developing measures for attenuating the impact of the COVID‐19 crisis while administering cranial SRS/SRT treatment in radiotherapy departments.

## 5 CONCLUSIONS

Based on retrospective investigations using the ExacTrac system database, the position error for patient motion using a thermoplastic mask system and a surgical mask together was found to be small. During the ongoing pandemic, a thermoplastic mask with a surgical mask is a feasible immobilization technique for brain SRS/SRT patients using the ExacTrac system.

## CONFLICT OF INTEREST

The authors declare that they have no conflict of interest to disclose.

## Data Availability

The data that support the findings of this study are available from the corresponding author upon reasonable request.
